# Mechanisms of skeletal muscle aging: insights from *Drosophila* and mammalian models

**DOI:** 10.1242/dmm.012559

**Published:** 2013-10-02

**Authors:** Fabio Demontis, Rosanna Piccirillo, Alfred L. Goldberg, Norbert Perrimon

**Affiliations:** 1 Department of Genetics, Harvard Medical School, Boston, MA 02115, USA; 2 Department of Developmental Neurobiology, Division of Developmental Biology, St Jude Children’s Research Hospital, Memphis, TN 38105, USA; 3 Department of Cell Biology, Harvard Medical School, Boston, MA 02115, USA; 4 Department of Oncology, IRCCS – Mario Negri Institute for Pharmacological Research, 20156 Milano, Italy; 5 Howard Hughes Medical Institute, Harvard Medical School, Boston, MA 02115, USA

## Abstract

A characteristic feature of aged humans and other mammals is the debilitating, progressive loss of skeletal muscle function and mass that is known as sarcopenia. Age-related muscle dysfunction occurs to an even greater extent during the relatively short lifespan of the fruit fly *Drosophila melanogaster*. Studies in model organisms indicate that sarcopenia is driven by a combination of muscle tissue extrinsic and intrinsic factors, and that it fundamentally differs from the rapid atrophy of muscles observed following disuse and fasting. Extrinsic changes in innervation, stem cell function and endocrine regulation of muscle homeostasis contribute to muscle aging. In addition, organelle dysfunction and compromised protein homeostasis are among the primary intrinsic causes. Some of these age-related changes can in turn contribute to the induction of compensatory stress responses that have a protective role during muscle aging. In this Review, we outline how studies in *Drosophila* and mammalian model organisms can each provide distinct advantages to facilitate the understanding of this complex multifactorial condition and how they can be used to identify suitable therapies.

## Introduction

After the age of 60 years, there are marked changes in multiple tissues and organs that together comprise the geriatric syndrome of frailty in humans. This condition is defined by decreased ability of the organism to withstand physical challenges and homeostatic perturbations (Fried et al., 2004). Skeletal muscle is a key reservoir of amino acids that sustain protein synthesis in other tissues, and limited muscle mass often associates with impaired responses to both stress and critical illness (Wolfe, 2006). After the age of 30 years, about 0.5–1% of muscle mass is lost per year in humans, with a dramatic acceleration of the rate of decline after the age of 65 years (Nair, 2005). The progressive loss of skeletal muscle mass and function in the aged is an important aspect of frailty that is often referred to as sarcopenia (Cruz-Jentoft et al., 2010), and it is largely responsible for the weight loss, weakness and impaired locomotion observed in the elderly. In addition, muscle-derived cytokines and growth factors (myokines) might also contribute to frailty by influencing metabolic homeostasis and systemic aging (Demontis et al., 2013).

**Fig. 1.**
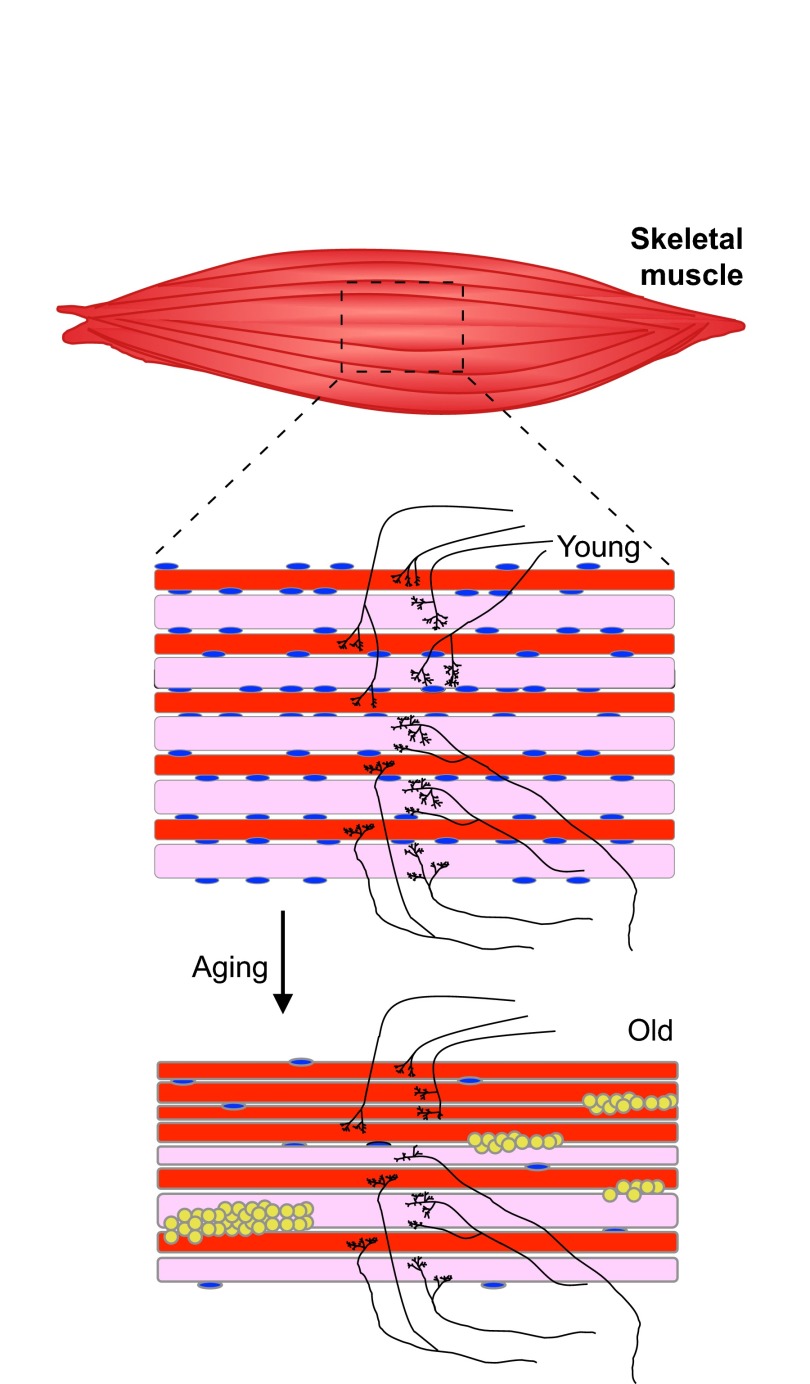
**Morphological changes in skeletal muscles during aging in mammals.** Muscle aging is characterized in mammals by a decline in the regenerative capacity, caused by a reduction in the number and function of muscle satellite cells (shown in blue). A decrease in the overall muscle strength and mass due to a decrease in the number and size of type IIb fibers (pink) and, to a lesser extent, type I fibers (red), is accompanied by defects in neuromuscular junctions and innervation (black). As the muscle ages, cycles of denervation and re-innervation eventually lead to changes in fiber-type composition, with a proportional increase in type I fibers (red), and grouping (stacking of red fibers). An accumulation of interstitial adipocytes (yellow) and decreased capillarization (not shown) are also observed. In *Drosophila*, defects in neuromuscular junctions have been described but there is currently no evidence for the presence of age-related changes in muscle mass that are seen in mammals.

Muscles are composed of different types of muscle cells (myofibers) that are classified as slow-twitch (type I) and fast-twitch (type IIb) fibers according to the mode of metabolism employed: slow-twitch fibers use aerobic (oxidative phosphorylation) respiration, whereas fast-twitch fibers utilize anaerobic metabolism (glycolysis) (Schiaffino and Reggiani, 2011). In both sarcopenia and cancer cachexia, glycolytic type IIb muscle fibers are smaller and are preferentially lost (Marzetti et al., 2009), whereas oxidative type I fibers are more commonly lost in obese individuals (Hickey et al., 1995). Myofiber loss can be accompanied by inflammation (Schaap et al., 2006), the infiltration of adipose tissue (Vettor et al., 2009), fibrosis (Rice et al., 1989) and decreased capillarization (Coggan et al., 1992; Degens, 1998). In addition to morphological changes in muscle (Fig. 1), sarcopenia is associated with several organism-wide changes, including an increase in the amount of adipose tissue (Stenholm et al., 2008). How the mechanisms causing sarcopenia are related to those involved in rapid muscle atrophy (Bonaldo and Sandri, 2013; Piccirillo et al., 2013) is an unresolved question. In young and old individuals, muscle mass is rapidly lost as a result of disuse, multiple systemic diseases or fasting. By contrast, sarcopenia is a slow process, in which some muscle mass is lost every year after adulthood, and this process becomes marked in humans older than 60 years.

Although the maintenance of both muscle mass and strength is needed for optimal performance, decrements in maximal strength are three times greater than the decline in muscle mass during human aging (Goodpaster et al., 2006; Metter et al., 1999), suggesting that muscle mass and strength are independently regulated. Moreover, muscle strength is a better predictor of mortality (related to any cause) during aging (Metter et al., 2002), suggesting that muscle function is a more important health parameter than muscle mass. Similar to what is observed in humans, the maximum isometric force (an indicator of muscle strength) decreases more than muscle mass during aging in mice, even when expressed relative to the cross-sectional area of the muscle (Faulkner et al., 1995; González and Delbono, 2001). Thus, the age-related loss of muscle strength cannot be solely explained by the loss of muscle mass: both muscle ‘quantity’ and ‘quality’ decline in aged animals. Here, we discuss how studies in model organisms are increasing our understanding of the fundamental mechanisms of sarcopenia, which might provide a basis for the development of therapies in humans.

## *Drosophila* as an emerging model of muscle aging

The analysis of sarcopenia in mammals has provided important insights into the mechanisms responsible for the progression of this condition in humans. Rhesus monkeys are the model organisms with the highest genetic similarity to humans (Uno, 1997) and, given the relatively large size of their muscles, are advantageous for detecting the progressive and relatively small changes in muscle mass during aging. However, studies on sarcopenia have benefited from the shorter lifespan and the many genetic tools available in mice. Most of the intrinsic and extrinsic changes regulating muscle aging in humans have been observed also in rodents, indicating that mice and rats are close models of human sarcopenia. However, the high costs associated with housing rodents for the course of their lifespan (2–3 years), the limited number of animals that can be analyzed per condition and the inability to perform large-scale genetic screens *in vivo*, as routinely done in invertebrates, suggests that analyses in simple model organisms might provide important complementary insight into the etiology of sarcopenia.

In *Drosophila melanogaster*, the organization and metabolism of skeletal muscle fibers is similar to that in mammals (Taylor, 2006; Piccirillo et al., 2013). However, the muscles undergo more dramatic age-related deterioration, presumably due to the lack of satellite stem cells and limited capacity for muscle repair in this organism. During the short lifespan of fruit flies (∼2–3 months), defects in flight, climbing and locomotion become progressively evident (Grotewiel et al., 2005; Martinez et al., 2007; Miller et al., 2008). Specifically, decreased functional capacity of both the direct and indirect flight muscles [necessary for the control of wing position and wing flapping, respectively (Dickinson, 2006)] is reflected by a reduction in wing beat frequency, flight duration, flight activity and the percentage of flies able to fly (Miller et al., 2008). Similarly, age-related functional impairment of leg and jump muscles causes defects in walking, climbing and jumping (Grotewiel et al., 2005).

The decline in muscle function in *Drosophila* most likely reflects a decrease in muscle strength (muscle ‘quality’); neither an age-related decrease in total muscle mass (muscle ‘quantity’) nor disuse atrophy or other types of wasting have been reported in adult flies (Piccirillo et al., 2013). The lack of studies about age-related changes in muscle mass presumably derives from the relatively small size of *Drosophila* muscles and the consequent difficulties in detecting small changes in muscle mass during aging. Studies are needed to assess whether a general decrease in muscle mass occurs during *Drosophila* aging and whether endocrine changes influence muscle aging in *Drosophila* as they do in mammals. Despite the current paucity of studies on these features of sarcopenia in *Drosophila* and possible differences with mammalian models, *Drosophila* could provide important insights into the intrinsic mechanisms regulating age-related muscle dysfunction, which seem to be largely conserved across species (see below). Studies in *Drosophila* are favored by the availability of an extensive genetic toolkit that enables genome-wide, muscle-specific interrogation of gene function during normal aging and in response to pharmacological, dietary and environmental interventions. Large cohorts of flies of a given age and genotype can be easily analyzed using several experimental approaches. Moreover, whereas aged rodents are housed in small laboratory cages that limit their physical activity, fruit flies can be housed in proportionally larger cages that allow the maintenance of activities and behaviors closer to those of populations in the wild. Because of these characteristics, *Drosophila* is emerging as a useful model organism to study muscle aging in concert with mammalian models. Here, we provide a comprehensive overview of the multifactorial origin of sarcopenia as gleaned through studies in mammals and, more recently, *Drosophila*, with particular emphasis on the role of protein homeostasis and stress responses.

## Extrinsic factors influencing sarcopenia

In humans, sarcopenia develops via multiple mechanisms. Age-related changes intrinsic to skeletal muscle seem to contribute to the decrease in muscle mass and strength; however, many extrinsic factors are also important. For example, aged organisms typically have lower nutrient intake, physical activity and anabolic hormone levels (including androgens, growth hormones and IGF1) than younger organisms, in addition to showing neuronal loss (i.e. fiber denervation) and decreased regenerative capacity (i.e. satellite stem cell dysfunction). Sarcopenia also enhances susceptibility to various highly catabolic diseases (e.g. cancer, cardiac failure, [chronic obstructive pulmonary disease], spinal injury) that are also more prevalent in the aged population. In turn, pharmacological treatments for cancer and other age-related diseases often induce muscle weakness as a side effect (Gilliam and St Clair, 2011; Hanai et al., 2007), thus aggravating sarcopenia. Here, we describe some of the endocrine changes and external (exogenous) factors that influence the progression of sarcopenia in mammals and *Drosophila*.

## Endocrine regulation of sarcopenia

Muscle atrophy in juvenile mammals seems to be largely reversible. For example, muscle loss upon fasting is rapidly and completely reversed upon re-feeding. Similarly, interventions such as exercise allow recovery from disuse atrophy in the young. However, such interventions are less effective in older individuals (Thomas, 2007). Changes in the endocrine environment during aging presumably contribute to the progression and limited reversibility of sarcopenia. Consistent with this, production of IGF1 and other anabolic cytokines in muscle tissue declines during aging (Goldspink and Harridge, 2004), which could explain the reduced synthesis of myofibrillar (e.g. myosin heavy chain) and mitochondrial proteins with age (Nair, 2005). Muscle-specific IGF1 overexpression attenuates the age-related loss of muscle mass (Barton-Davis et al., 1998; Musarò et al., 2001). However, long-term administration of IGF1, even if effective, might not be an appropriate treatment for sarcopenia and cachexia because insulin-IGF1 signaling has been shown to promote cancer growth and to shorten lifespan (Kenyon, 2010).

Aged muscles are also less responsive to anabolic and catabolic stimuli than are young muscles. Exercise and ingestion of amino acids normally stimulate protein synthesis in human muscles but are less effective at doing this in the elderly, a phenomenon termed ‘anabolic resistance’, which likely contributes to sarcopenia (Breen and Phillips, 2011). Among the catabolic hormones, the glucocorticoids (steroids normally produced in the adrenal cortex but widely administered medically) have a primary role in mobilizing amino acids from muscle proteins to favor gluconeogenesis during fasting. Glucocorticoids inhibit muscle protein synthesis and enhance proteolysis. In catabolic states, they help induce expression of atrogin-1 and MuRF1, atrophy-related E3 ubiquitin ligases that accelerate protein turnover via the ubiquitin proteasome system (UPS) (reviewed in Bonaldo and Sandri, 2013; Piccirillo et al., 2013). However, glucocorticoids have a reduced catabolic effect in muscles from aged sarcopenic rats and fail to enhance protein breakdown (Altun et al., 2010; Dardevet et al., 1995). This finding is unexpected because sarcopenia might have been explained by greater sensitivity of the aged to catabolic factors (as occurs in low-insulin states). The cellular basis for altered sensitivity to anabolic and catabolic stimuli in aging and the physiological consequences of this reduced sensitivity to extrinsic factors remain unclear.

## Satellite stem cells and muscle repair

Satellite cells are progenitor cells involved in muscle repair after injury and perhaps in the maintenance of muscle mass (Biressi and Rando, 2010). Although the loss of muscle mass in aged mammals can be compensated for by new fiber formation, the regenerative capacity of muscles decreases progressively during aging (Vinciguerra et al., 2010). Specifically, both the number and regenerative ability of satellite cells decline during aging (Fig. 1) (Shefer et al., 2010) because of age-related changes in endocrine factors that alter their myogenic potential (Brack et al., 2007). In addition, with aging, satellite cells exhibit decreased responsiveness to endocrine and paracrine stimuli that normally induce their activation and proliferation (Gopinath and Rando, 2008; Kuang et al., 2008). For example, in old mice (around 2 years of age), the satellite cell niche (i.e. the fibers adjacent to the satellite cells) expresses lower levels of the Notch ligand Delta-like 1, which is required for the activation and proliferation of satellite cells (Conboy and Rando, 2002). In addition, higher levels of fibroblast growth factor 2 (FGF2) during aging lead to the loss of quiescence and self-renewal capacity (Chakkalakal et al., 2012). Cross-transplantation and parabiotic studies in which the stem cells from young mice are exposed to factors present in old animals (and vice versa) further indicate that the behavior of stem cells depends largely on cytokines and hormones that differ markedly in the aged, such as IGF1 (Jones and Rando, 2011; Carlson and Faulkner, 1989). Moreover, dysfunctional interactions of satellite cells with muscle connective tissue fibroblasts and infiltrating macrophages and neutrophils, which normally promote muscle repair after injury, presumably contribute to decreased muscle regeneration in aged organisms (Carosio et al., 2011). Altogether, these results suggest that changes in the extracellular environment are important determinants of regenerative capacity in the aged.

Along with endocrine and paracrine stimuli, intrinsic defects in satellite cells might play a role in skeletal muscle aging. For example, the decreased maintenance of telomere length in Ku80 heterozygous mutant mice, which are defective in DNA double-strand-break repair, diminishes stem cell renewal and accelerates muscle aging (Didier et al., 2012). However, whether satellite cells contribute to the turnover of normal uninjured adult muscles is largely unknown. Both calorie restriction (CR) and exercise not only delay muscle aging but also enhance the repair activity of satellite cells in mice (Cerletti et al., 2012; Shefer et al., 2010), suggesting that satellite cells might contribute to the protective responses induced by these interventions.

Muscle stem cells have not been identified in adult insects. This suggests that their skeletal muscles lack the capacity for regeneration during aging. This fundamental difference could account for the more obvious morphological and functional changes in aging *Drosophila* muscle compared with mammals, and is presumably related to *Drosophila*’s short lifespan.

## Defects in the neuromuscular junction and denervation

In addition to changes in endocrine signals and reduced regenerative capacity, age-related defects in neuromuscular junctions (NMJs) and death of motor neurons (Fig. 1) can lead to chronic cycles of denervation and re-innervation, especially of type IIb fast-twitch fibers. Denervation contributes to loss of muscle mass in humans and rodents (Delbono, 2003; Jang and Van Remmen, 2011). Because muscle fiber type is largely determined by the type of innervation and thus by contractile activity, re-innervation of type IIb fast-twitch fibers leads to fiber-type switch if it occurs via axonal sprouting from nerves that innervate adjacent slow-twitch fibers. Moreover, type I and II fibers cluster into distinct groups (fiber-type grouping) after re-innervation rather than forming the normal ‘checkerboard’ pattern, where type I and type II fibers intermix. If re-innervation is insufficient, then fibers can undergo atrophy or apoptosis (Larsson and Ansved, 1995).

Age-related defects in the NMJ and innervation have also been reported in *Drosophila* (Beramendi et al., 2007). Specifically, NMJ bouton size increases and the length and diameter of nerve branches decrease during aging (Beramendi et al., 2007). Moreover, synaptic transmission along the giant fiber neuronal circuit decreases during aging, and indirect flight muscles become unresponsive to stimulation (Martinez et al., 2007). Altogether, in some fibers, changes in NMJs and denervation clearly contribute to muscle aging in *Drosophila* and mammals, although the specific effect on contractile performance is unknown.

## Intrinsic defects leading to loss of muscle function during aging

**Fig. 2.**
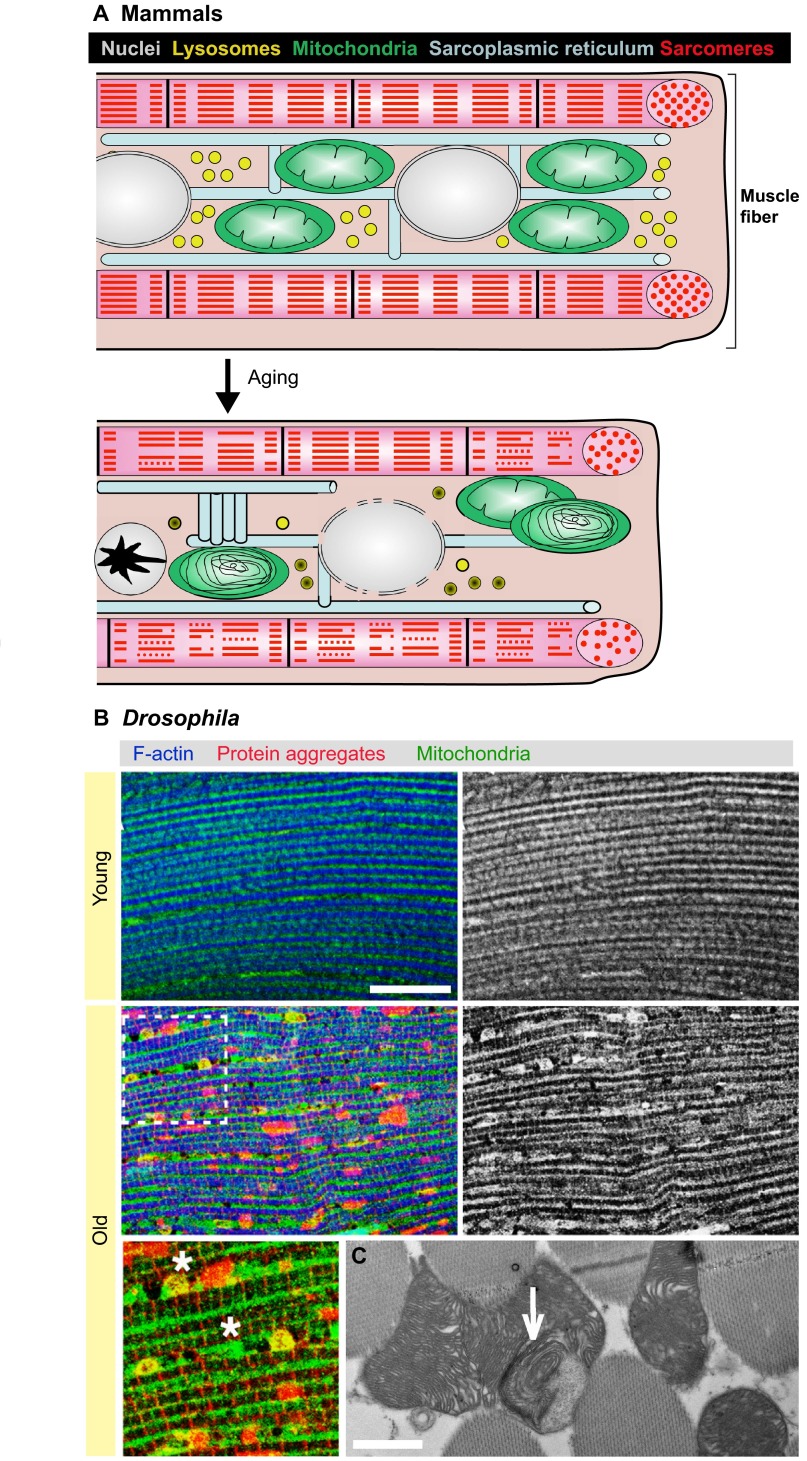
**Intracellular changes in muscle fibers during aging in mammals and *Drosophila***. (A) During aging, mammalian muscle fibers progressively accumulate damaged proteins in most cellular compartments, including the sarcomeres (red), which show loss of organization and structure (myofibril is shown in pink). Lysosomes (yellow) accumulate lipofuscin deposits and have decreased capability for degradation, resulting in improper turnover of organelles. Abnormalities affecting mitochondria (green) include decreased respiratory capacity, increased loss, abnormal size and irregular distribution. Misfolding and aggregation of membrane proteins of the sarcoplasmic reticulum (blue) can result in tubular aggregates and improper calcium handling in old age. Nuclei (gray) have DNA damage and epigenetic changes, loss of nuclear membrane integrity (due to damage of nuclear pore components), and can be lost during syncytial apoptosis (black star), which can result in changes in myofiber size without myofiber death. Similar intracellular changes, apart from changes in myofiber size, have also been described in *Drosophila* muscles during aging (not shown). (B) Staining of *Drosophila* indirect flight muscles from young (1 week old) and old (8 weeks old) flies with antibodies for Sod2 (mitochondria; green), poly-ubiquitin (protein aggregates; red), and phalloidin (F-actin, a component of sarcomeres; blue). *Drosophila* muscles display extensive protein damage and organelle dysfunction in old age. The number and function of lysosomes declines during aging and strikingly results in the accumulation of poly-ubiquitin protein aggregates. Although mitochondria (green) are homogeneously distributed along myofibrils (blue) in young age, they are enlarged (*) or absent from certain areas of the fiber in old age. The panel in the bottom left corner is a zoomed version of the section marked by the dashed line box directly above. (C) A transmission electron micrograph of *Drosophila* indirect flight muscles from old flies highlights the presence of mitochondria with ‘swirls’ of abnormally arranged inner cristae (arrow). These defects are normally not seen in young flies (not shown). Scale bar: 10 μm (B); 1 μm (C). See the main text for a more comprehensive description of the age-related cellular changes in mammalian and *Drosophila* skeletal muscles.

Mammalian muscles display a number of intrinsic alterations during aging (Fig. 2A), which reduce functional capacity together with extrinsic factors (Brooks and Faulkner, 1994; Nair, 2005). Muscles of aged mammals show fiber atrophy (Fujisawa, 1974; Fujisawa, 1975; Tomonaga, 1977), increased apoptosis (Marzetti and Leeuwenburgh, 2006), DNA damage (Aiken et al., 2002; Szczesny et al., 2010), reduced protein synthesis (Hasten et al., 2000; Yarasheski et al., 1999), age-related decline in autophagic degradation (Wohlgemuth et al., 2010), lysosomal dysfunction (accumulation of lipofuscin deposits) (Beregi et al., 1988; Hütter et al., 2007), accumulation of advanced glycation end-products (Snow et al., 2007), insoluble polyubiquitylated proteins (Yamaguchi et al., 2007), increased heterochromatin marks (Kreiling et al., 2011), changes in microRNA expression (Drummond et al., 2011), and altered nuclear shape and spatial disorganization of nuclei (Cristea et al., 2010).

A number of age-related intrinsic changes in skeletal muscle (Fig. 2B) have also been reported in *Drosophila* and other insects (Takahashi et al., 1970; Webb and Tribe, 1974). These and additional studies discussed below have highlighted ultrastructural changes in most cellular organelles of muscle fibers during aging. Age-related defects in the autophagy-lysosome system, exemplified by the accumulation of cytoplasmic p62–poly-ubiquitin protein aggregates (Demontis and Perrimon, 2010), could underlie improper turnover of dysfunctional cellular components and organelles. Together with DNA damage (Garcia et al., 2010) and reduced protein synthesis (Webster et al., 1980), these defects presumably limit the capacity for replacement of faulty cellular components, and are a likely cause of the increased functional defects and apoptosis observed in aged *Drosophila* muscles (Zheng et al., 2005).

The similar morphological changes detected in myofibers of *Drosophila* and mammals in old age suggest commonality in the mechanisms leading to muscle functional decay at the cellular level. We describe below in greater detail the alterations that occur with aging in a few cellular organelles and their likely significance in sarcopenia.

## Decline in mitochondrial function and metabolic homeostasis with age

Several defects are observed in mitochondria isolated from aged skeletal muscles from mice and humans. These include changes in mitochondrial enzyme concentrations (Staunton et al., 2011), decreased mitochondrial protein synthesis (Rooyackers et al., 1996), dysfunction of the mitochondrial permeability transition pore (Seo et al., 2008), mitochondrial enlargement that could indicate increased fusion (Terman and Brunk, 2004), increased mitochondrial DNA mutations (Lee et al., 1997; McKenzie et al., 2002; Melov et al., 1995; Wanagat et al., 2001), and greater generation of reactive oxygen species (ROS) (Mansouri et al., 2006). Decreased function and altered stoichiometry of mitochondrial proteins is in turn thought to be responsible for lower respiratory capacity (Chabi et al., 2008; Trounce et al., 1989) and a decrease in mitochondria and ATP levels (Ljubicic et al., 2009; Short et al., 2005) in the aged. Additionally, defects in mitochondrial iron homeostasis might underlie the age-related accumulation of intracellular iron observed in rats, which in turn increases oxidative stress (Marzetti et al., 2009). Loss of mitochondrial function contributes to decreased fatty acid metabolism (Tucker and Turcotte, 2002; Houtkooper et al., 2011) and thereby the intracellular accumulation of lipids (a prime metabolic substrate of mitochondria) (Crane et al., 2010; Pugh et al., 2013), which in turn promotes insulin resistance in aged individuals (Eckardt et al., 2011). In agreement with this model, targeting peroxisomal catalase to mitochondria can preserve mitochondrial function and reduce oxidative damage and lipid-induced insulin resistance in mice (Lee et al., 2010a). Conversely, mitochondrial superoxide dismutase 2 (SOD2) deficiency leads to protein damage and metabolic alterations that could contribute to the loss of muscle strength during aging (Lustgarten et al., 2009; Lustgarten et al., 2011).

It should be noted that the majority of studies examining mitochondrial function during skeletal muscle aging involved purified mitochondria; therefore, the functional changes observed do not necessarily reflect those occurring in the normal physiological environment. Indeed, analyses of mitochondrial function in permeabilized fibers suggest that mitochondrial respiratory function and capacity to oxidize fatty acids are not significantly altered in skeletal muscles during aging (Hütter et al., 2007; Picard et al., 2010). However, this was refuted in a recent study that provided evidence for age-related mitochondrial dysfunction in permeabilized myofibers (Lanza et al., 2012). Differences in the experimental settings could explain discrepancies between studies involving permeabilized myofibers. Altogether, the results suggest that mitochondrial defects and oxidative stress could contribute to metabolic dysfunction in the aged.

Although mitochondrial dysfunction and oxidative protein damage are more common in aerobic than in glycolytic fibers because of the high mitochondrial content of the former (Choksi et al., 2008), other age-related metabolic defects might underlie the functional decline of glycolytic fibers, including increased glycolysis, and decreased glucose uptake and glycogen synthesis (Houtkooper et al., 2011), which are typically associated with insulin resistance in aged humans (Cline et al., 1999). Other metabolites and metabolic pathways, such as those that regulate amino acid synthesis and degradation, are also altered during muscle aging in mice (Houtkooper et al., 2011) and could play a role in age-related muscle functional decline.

Mitochondrial respiratory functions also undergo age-related decline in *Drosophila* (Ferguson et al., 2005). Indeed, several mitochondrial genes and metabolic enzymes involved in oxidative phosphorylation and the tricarboxylic acid cycle are downregulated in aged *Drosophila* (Schwarze et al., 1998; Girardot et al., 2006). Electron microscopy images show mitochondrial enlargement and aberrant organization (‘swirls’) of the mitochondrial inner cristae in aged muscles (Fig. 2C) (Takahashi et al., 1970; Webb and Tribe, 1974), features that also occur in response to hyperoxia (Walker and Benzer, 2004) and have been observed also in flight muscles from aged blowflies (Sacktor and Shimada, 1972). Additional age-related changes include mitochondrial protein and DNA damage (Toroser et al., 2007; Yui et al., 2003), and lipid peroxidation (Magwere et al., 2006). Some of these defects might arise as a consequence of oxidative stress during aging. Consistent with this, flies with mutations in the FOXO target gene encoding the antioxidant protein Sestrin have dysfunctional mitochondria and show increased ROS production in muscles (Lee et al., 2010b), similar to the defects observed in older wild-type flies (Takahashi et al., 1970). In addition, aging increases the production of hydrogen peroxide in muscle mitochondria of *Drosophila* (Cochemé et al., 2011) and houseflies (Sohal and Sohal, 1991). Because hydrogen peroxide reacts with proteins, lipids and DNA, leading to oxidative modification of these cellular constituents, it might affect several cellular functions and thus regulate tissue aging and lifespan. However, hydrogen peroxide levels are not consistently altered by interventions that modulate lifespan in flies (Cochemé et al., 2011), and do not correlate with the different degree of atrophy observed in type I and type II myofibers in mice (Picard et al., 2011). Further complexity in interpreting the role of oxidative stress during aging derives from the observation that, although high levels of ROS are detrimental, moderate ROS production can induce signal transduction pathways that mount protective stress responses that extend lifespan in invertebrates and mice (Gems and Partidge, 2013). Altogether, these studies indicate an important and complex role of mitochondrial dysfunction, oxidative stress and metabolic changes during skeletal muscle aging in *Drosophila* and mammals.

## Changes in sarcomeres during aging

The sarcomere is the basic repeating contractile subunit composing the myofibrils (the contractile apparatus of myofibers) in skeletal muscles. Muscle contraction results from the ATP-dependent sliding of sarcomeric myosin-based thick filaments over actin-based thin filaments. In mice and humans, age-related structural and functional alterations in sarcomeric proteins might explain the decline in force generation that is typical of old age (Prochniewicz et al., 2007). Studies with permeabilized fibers and *in vitro* motility assays indeed highlight age-related intrinsic defects in the function of contractile proteins during aging, including a reduction in actomyosin ATPase activity (Lee et al., 2010b; Prochniewicz et al., 2007). One study demonstrated that myosin from muscles of aged rodents and humans are less able to move actin than myosin taken from the muscles of younger animals (Höök et al., 2001). Oxidative modifications could induce structural changes that underlie the functional decline of contractile proteins during muscle aging (Perkins et al., 1997; Lowe et al., 2001). Decreased expression of sarcomeric proteins and changes in the expression of myosin isoforms are also likely to be involved in muscle aging in mice (Larsson et al., 1997; Nair, 2005).

In *Drosophila*, sarcomere-related defects have also been reported, including changes in myofibrillar protein composition and function (Miller et al., 2008), and decreased length and increased disorganization of the sarcomeres (Takahashi et al., 1970; Webb and Tribe, 1974).

In mice, several transcription factors [such as serum response factor (SRF)] and the autophagy adaptor proteins p62 (SQSTM1) and NBR1 localize to sarcomeres (Braun and Gautel, 2011), presumably to enable these factors to respond to changes in contractile activity. Therefore, in addition to influencing muscle contraction, the age-related damage and disorganization of sarcomeres could trigger changes in the localization and activity of sarcomere-associated signaling factors, which might in turn induce changes in other cellular compartments. This hypothesis remains to be tested.

## The sarcoplasmic reticulum and calcium handling

A plethora of ultrastructural changes occur in murine muscles with aging, including the accumulation of tubular aggregates of sarcoplasmic reticulum (SR) membranes (Schiaffino, 2012). This phenomenon can lead to abnormal Ca^2+^ storage and release from the SR (Jiménez-Moreno et al., 2008; Russ et al., 2011). Additional molecular changes during mammalian aging are associated with a decline in Ca^2+^ homeostasis, including decreased levels of synaptophysin mitsugumin-29 (Zhao et al., 2008) and muscle-specific inositide phosphatase (MIP; also known as MTMR14) (Romero-Suarez et al., 2010), as well as functional changes in the dihydropyridine receptors, which are L-type voltage-dependent Ca^2+^ channels (Payne et al., 2004). These channels normally interact with the ryanodine receptor 1 (RyR1) to promote Ca^2+^ release from the SR after membrane depolarization in transverse tubules. In the cytosol, SR-released Ca^2+^ ions bind to troponin C, triggering formation of actomyosin cross-bridges, shortening of sarcomeres and force development. RyR1 receptors are damaged in aging muscles because of oxidation and nitrosylation of their cysteine residues (Andersson et al., 2011), which results in remodeled RyR1 complexes devoid of the channel-stabilizing subunit calstabin 1, which normally limits the open state of RyR1 channels. Consequently, cytoplasmic Ca^2+^ leaks from the SR, impairs excitation-contraction coupling and causes muscle weakness (Andersson et al., 2011). Andersson et al. further showed that a small molecule, S107, promotes the association of calstabin with the RyR1 complex, inhibits Ca^2+^ leaks in aged animals, and thus restores muscle force and exercise capacity (Andersson et al., 2011). The therapeutic potential of this recently discovered small molecule is clearly important to define.

Disorganization of the SR has been observed also in old fruit flies (Takahashi et al., 1970); therefore, *Drosophila* might provide insight into the mechanisms of age-related deterioration of this cellular compartment. However, insect indirect flight muscles have a less extensive SR than mammalian muscle (Dickinson, 2006). In addition, in flies, muscle contraction does not depend on depolarization-induced Ca^2+^ release from the SR but is rather mechanically activated by stretching and inactivated by fiber shortening, with Ca^2+^ levels being needed for maintaining the muscle in a permissive state for contraction and for adjusting power output (Gordon and Dickinson, 2006). Therefore, the applicability of flies for the study of Ca^2+^ leaking from the SR is limited because of these differences with mammals.

## Nuclear and plasma membrane integrity

Muscle membranes are susceptible to damage caused by the mechanical stress of contraction. In mammals, the membrane-associated network of surface proteins known as dystrophin glycoprotein complex (DGC) helps maintain plasma membrane integrity by stabilizing the muscle membrane and limiting any physical damage caused by contraction. If membrane tears are generated, components of the ferlin family of proteins, including dysferlin and myoferlin, and their interacting partners (mitsugumin-53, caveolin-3, annexin A1 and others) accumulate at the site of membrane disruption, and function as Ca^2+^ sensors to regulate resealing (Doherty and McNally, 2003). Null mutations in the dystrophin and dysferlin genes cause early onset of Duchenne or limb-girdle muscular dystrophies, respectively, in mammals, whereas hypomorphic mutations in these genes might affect plasma membrane stability and repair in old age (Doherty and McNally, 2003). Furthermore, ferlin proteins might also mediate the fusion of satellite cells to pre-existing fibers to maintain muscle mass after damage or in old age, as suggested by the requirement of myoferlin for both myoblast-myoblast and myoblast-myotube fusion during mouse development (Doherty et al., 2005). *Drosophila* is a potentially useful system for analyzing muscle membrane repair during aging because only one myoferlin and dysferlin homolog (*misfire*) is present in flies, unlike rodents.

As in humans, mutations in the *Drosophila* homologs of the DGC components result in muscle degeneration, mobility defects and shortening of lifespan (Shcherbata et al., 2007). Interestingly, a recent genetic screen for interactors of the DGC in *Drosophila* muscles identified several genes involved in stress resistance (Kucherenko et al., 2011). These included the genes *βν-integrin*, *Fhos*, *capt* and *CG34400*, which mediate the reorganization of the cytoskeleton in response to mechanical stress. Taken together, these findings suggest that defects in the DGC complex and plasma membrane integrity could contribute to muscle functional loss with aging.

Nuclear membranes are also susceptible to age-related deterioration. In particular, aging is characterized in mice and *Drosophila* by an increase in the number of muscle nuclei having aberrant shape, condensed chromatin and spatial disorganization (Brandt et al., 2008; Cristea et al., 2010). The nuclear lamina, which maintains the shape and mechanical stability of the nucleus, and nuclear pore complexes (NPCs), which mediate the transport of molecules across the nuclear envelope, are thought to be particularly sensitive to age-related damage. Specifically, the scaffold nucleoporins Nup107-Nup160 of NPCs persist for the entire lifespan of post-mitotic cells and are vulnerable to oxidative modifications that increase nuclear permeability during aging (D’Angelo et al., 2009). In addition, altered shape and function of the nuclear envelope, as observed in old age, can be induced in young age by overexpression of Kugelkern and Lamin B, two farnesylated lamina proteins regulating nuclear stability (Brandt et al., 2008), and by production of permanently farnesylated mutant Lamin A (also known as LMNA), which leads to Hutchinson-Gilford progeria, a syndrome characterized by premature and accelerated aging, musculoskeletal degeneration and muscle weakness (Prokocimer et al., 2013). Interestingly, flies carrying *lamin* mutations have premature locomotor defects (Lenz-Böhme et al., 1997; Muñoz-Alarcón et al., 2007), as also observed in flies overexpressing *kugelkern* and *lamin B* in muscle (Brandt et al., 2008). Thus, some of the nuclear defects responsible for progeria syndromes could also affect the loss of muscle function in old age.

## Stress responses and protein quality control in muscles during aging

A general feature of aging is the induction of multiple stress responses (Haigis and Yankner, 2010). Although some of the age-related changes in muscle are thought to contribute to sarcopenia, others could be compensatory protective responses that are activated to cope with aging-associated protein damage. In line with this, heat shock proteins, DNA repair genes and oxidative stress resistance pathways are activated in the muscles of aging mice (Park and Prolla, 2005). Similarly, analysis of gene expression changes in *Drosophila* muscles during aging has highlighted increased expression of components of the 26S proteasome and the induction of antioxidant stress responses. These changes include increased transcription and translation of the major cytosolic chaperone Hsp70 (Wheeler et al., 1995), upregulation of the JNK pathway, and increased expression of the *ferritin*, *glutathione transferase D1* and *metallothionein A* genes (Girardot et al., 2006). This gene expression pattern resembles that occurring after oxidative stress in *Drosophila* (Pickering et al., 2013). Moreover, studies in rats have shown that, during sarcopenia, there is increased expression of the chaperone-dependent ubiquitin ligase CHIP (Altun et al., 2010), which catalyzes the proteasomal degradation of misfolded proteins. The induction of this protein probably represents a protective mechanism to cope with the progressive accumulation of damaged proteins during aging. In fact, CHIP knockout mice have shorter lifespans and undergo accelerated muscle mass loss during aging (Min et al., 2008). Also expressed at increased levels in the muscles of aged rats is the p97-VCP ATPase complex and its cofactors, which promote the degradation of ubiquitylated misfolded proteins in the ER and cytosol (Altun et al., 2010). The expression of the major molecular chaperones (Hsp70 and Hsp90) also increases during muscle aging in mice (Clavel et al., 2006; Ferrington et al., 2005). These chaperones selectively bind misfolded proteins and promote their refolding or hydrolysis. However, work in *Drosophila* shows that Hsp70 overexpression does not prevent the age-related accumulation of poly-ubiquitin protein aggregates, although Hsp70 associates with them and presumably reduces their toxicity by avoiding their interaction with native proteins (Demontis and Perrimon, 2010). Similar to *Drosophila*, detergent-insoluble fractions of aged muscles in humans and mice contain elevated levels of poly-ubiquitylated proteins (Yamaguchi et al., 2007; Hwee et al., 2013). Moreover, the insoluble levels of Hsp27 and αB-crystallin also increase during muscle aging (Yamaguchi et al., 2007), suggesting a role for these chaperones in decreasing the proteotoxicity of poly-ubiquitylated misfolded proteins.

Altogether, these findings highlight a number of adaptations of older muscles to cope with misfolded proteins. By contrast, the data for the expression of the E3 ubiquitin ligases that are characteristic of rapid atrophy in younger animals are less conclusive: levels of atrogin-1 do not change, whereas levels of another ligase, MuRF1, rise in rat muscles during sarcopenia (Altun et al., 2010). Reports on the regulation of E3 ubiquitin ligases during sarcopenia in mice are also inconsistent (Clavel et al., 2006; Gaugler et al., 2011). The amounts of critical ligases are known to rise and then fall during rapid atrophy in mouse muscles (Sacheck et al., 2007), but detailed time courses of such events during sarcopenia have not been obtained. In addition, most studies only monitor mRNA but not enzyme levels. Interestingly, recent findings indicate that atrogin-1 knock-out mice are short-lived and experience higher loss of muscle mass during aging than control mice (Sandri et al., 2013), indicating that the activity of this E3 ubiquitin ligase is required to preserve muscle mass and function during aging in mice. Moreover, MuRF1 null mice experience a higher decay of muscle strength during aging than controls, although muscle mass is at least in part preserved in these mice (Hwee et al., 2013).

In *Drosophila* muscles, additional age-induced responses include increased expression of the mitochondrial chaperones Hsp22 and Hsp23, which are small heat shock proteins whose induction is likely to indicate the activation of the mitochondrial unfolded protein response (UPR^mt^) (Wheeler et al., 1995), a transcriptional stress response that deals with misfolded proteins in mitochondria. In mice, overexpression of the mitochondrial chaperone Hsp10 in skeletal muscle prevents age-related decline in muscle mass and strength (Kayani et al., 2010), providing further evidence that the UPR^mt^ is a protective response to sarcopenia. Moreover, analyses of mice with a reporter based on *Xbp1* mRNA splicing indicated that muscle aging is also characterized by the induction of the unfolded protein response in the endoplasmic reticulum (UPR^ER^) (Iwawaki et al., 2004). Moreover, other UPR^ER^ markers, including BiP, PDI and CHOP, are also upregulated during muscle aging in mice (Hwee et al., 2013). The UPR^ER^, which is induced in response to the accumulation of misfolded proteins in the ER, entails the phosphorylation of the translation initiation factor eIF-2α by PERK (protein kinase RNA-like endoplasmic reticulum kinase), which presumably contributes, together with decreased IGF1 levels, to the overall decline in muscle protein synthesis during aging in both mammals (Hasten et al., 2000; Yarasheski et al., 1999) and *Drosophila* (Webster et al., 1980). Although decreased protein synthesis can be deleterious and leads to atrophy, partial inhibition [as occurs during dietary restriction (DR) and in the unfolded protein response] is likely to be protective against certain stresses.

The UPR^ER^ is also activated in muscles during exercise via PGC-1α (peroxisome proliferator-activated receptor gamma coactivator 1α) and the transcription factor ATF6α (Wu et al., 2011; Mattson et al., 2000). PGC-1α also increases the expression of many genes involved in protein folding (including ER chaperones), energy production, mitochondrial biogenesis and defense against oxygen radicals, as well as NMJ components (Wu et al., 2011). Thus, this exercise-induced transcriptional coactivator seems to be important in overall protein quality control, which could contribute to its roles in enhancing exercise capacity and resistance to sarcopenia (Wu et al., 2011).

Despite the initial protection that stress responses can confer, they are a potential cause of muscle deterioration if excessive. In the muscles of old rats, there seems to be a prolonged decrease in protein synthesis and increase in protein degradation via the UPS, all of which might initially compensate for the accumulation of damaged proteins during aging (Clavel et al., 2006; Gaugler et al., 2011; Ludatscher et al., 1983; Wohlgemuth et al., 2010). However, excessive induction of some of these stress responses, including JNK signaling and UPS activity, is known to be deleterious and, in muscle, could promote degradation of functional myofibrillar proteins, lead to the loss of muscle bulk and trigger apoptosis.

Additional studies in *Drosophila* and mammals emphasize the importance of fine-tuning protein degradation pathways to maintain muscle mass and function. These studies have revealed that general activation of the UPS and induction of specific E3 ubiquitin ligases are crucial for rapid atrophy. However, the UPS is also required in all cells to selectively eliminate misfolded or damaged proteins, particularly under stressful conditions. Among the various regulators, FoxO transcription factors are key players in muscle protein homeostasis by virtue of their ability to activate multiple systems of protein disposal. Although overexpression of wild-type FoxO3A does not induce muscle atrophy in mice, overexpression of constitutively active FoxO3A triggers rapid loss of muscle mass, and activation of FoxO proteins seems to be crucial in multiple types of atrophy, in which it induces both the UPS and autophagy (Sandri et al., 2004; Zhao et al., 2007). However, moderate FoxO3A activity might have a protective role by promoting the preferential disposal of damaged proteins and organelles while avoiding the unselective loss of muscle mass. In agreement with this hypothesis, overexpression of wild-type FOXO in muscles of adult flies is protective because it prevents the age-related decline in protein homeostasis and preserves muscle function (Demontis and Perrimon, 2010). FOXO activates the autophagy-lysosome proteolytic system, which in turn decreases the age-related accumulation of p62–poly-ubiquitin protein aggregates in muscles. Conversely, during aging, short-lived *foxo*-null flies accumulate more protein aggregates in skeletal muscles than do wild-type flies (Demontis and Perrimon, 2010). Altogether, different levels of FOXO activity can be either detrimental or protective, and therefore lead to radically distinct outcomes on muscle protein homeostasis and age-related functional decay.

Further indications of the protective role of autophagy in cellular quality control is that ablation of the autophagy gene *Atg7* in mouse muscles results in the accumulation of p62–poly-ubiquitin protein inclusions, abnormal mitochondria and misalignment of sarcomeric Z-lines, all of which lead to muscle weakness (Masiero et al., 2009). Several adaptive responses are induced in *Atg7* knockout mice, including the upregulation of UPS components, atrogin-1, MuRF-1, the ER chaperone BiP (GRP78) and the UPR^ER^ (Masiero et al., 2009). Despite these compensations, loss of autophagy leads to a 40% decrease in the size of oxidative and glycolytic fibers. In addition, inhibiting autophagy aggravates the loss of muscle mass in response to denervation in mice (Masiero et al., 2009). Similarly, muscle-specific knockout of *Atg5* (another autophagy gene) also leads to loss of muscle mass and accumulation of protein aggregates in mice (Raben et al., 2008). Furthermore, sustained activation of mTORC1 signaling in mouse skeletal muscle is observed during aging (Sandri et al., 2013) and leads to autophagy inhibition and severe age-related muscle atrophy characterized by the accumulation of p62-containing protein aggregates and dysfunctional mitochondria (Castets et al., 2013). Interestingly, treatment with the weakly basic antimalarial drug chloroquine can also cause muscle weakness, atrophy, and muscle accumulation of β-amyloid species (similar to those observed in the brains of Alzheimer’s patients), which might be attributable to the inhibitory action of this drug on lysosomal proteolysis (Tsuzuki et al., 1995).

Although caspases and the Ca^2+^-dependent proteases calpains have long been thought to catalyze myofibril degradation in disease states, including sarcopenia (Combaret et al., 2009), it is now clear that sarcomeric components are degraded via the UPS both normally and during atrophy (Cohen et al., 2009; Cohen et al., 2012; Solomon and Goldberg, 1996; Piccirillo and Goldberg, 2012). The autophagy-lysosome system degrades most organelles and soluble cytoplasm but it is also required to maintain sarcomeric organization in *Drosophila* and mammals (Arndt et al., 2010; Masiero et al., 2009). For example, the co-chaperone Starvin (BAG-3 in mammals) together with Hsc70, the small heat shock protein HspB8, CHIP and the autophagic adaptor p62 mediate the degradation of some damaged sarcomeric proteins of the Z-disc (such as filamin) via selective autophagy to maintain sarcomere integrity in both *Drosophila* and mice (Arndt et al., 2010).

Specific recognition and disposal of damaged proteins while sparing native, undamaged ones is essential to preserve protein homeostasis and to avoid loss of muscle function. In mammalian cells and *Drosophila*, only the ubiquitin ligase CHIP has been shown to selectively catalyze the ubiquitylation of misfolded cytosolic proteins, which are recognized by their prolonged association with Hsp70 or Hsp90. However, other E3 ubiquitin ligases must also catalyze the selective degradation of harmful unfolded proteins, as observed in yeast. In addition to delaying normal aging, therapeutic interventions that preserve protein homeostasis might reduce the progression of inclusion-body myositis and other myopathies characterized by the age-related accumulation of protein inclusions (Askanas and Engel, 2002). Taken together, studies in *Drosophila* and mammals indicate an important role of protein quality control in preserving muscle function during aging and preventing age-related myopathies in humans.

## Genetic propensity to sarcopenia

Although most aged humans display some signs of muscle weakening, overt, debilitating sarcopenia affects only 35–45% of people. Genome-wide association studies (GWAS), whole-genome linkage studies, and gene expression and quantitative trait loci (QTL) mapping in both human populations and mammalian models are expanding our understanding of the genetic propensity to develop sarcopenia (reviewed in Tan et al., 2012).

Recent surveys indicate that polymorphisms in genes controlling muscle mass in humans and mammals (Bonaldo and Sandri, 2013; Piccirillo et al., 2013), including the genes encoding insulin growth factor 1 (IGF1), myostatin, follistatin and components of the activin receptor protein complex, are linked to increased risk of sarcopenia (Tan et al., 2012). Although it is currently unknown whether these polymorphisms increase or rather decrease the function of these regulators of muscle mass, molecular analysis and interrogation of other data might provide important information. For example, both polymorphisms in the vitamin D receptor gene (Roth et al., 2004) and low serum vitamin D levels have been implicated in sarcopenia (Visser et al., 2003) and in increased myofibrillar protein degradation at younger ages (Wassner et al., 1983), suggesting that polymorphisms in the vitamin D receptor probably lead to its partial inactivation.

Genotypes associated with athletic performance and muscle strength in young humans could also influence the progression of sarcopenia. The nonsense mutation R577X in the α-actinin-3 (*ACTN3*) gene, which is present in ∼18% of the world’s population (Yang et al., 2009), results in a deficiency of α-actinin-3, a component of the sarcomeric Z-disc in fast-twitch muscle fibers. *ACTN3* knockout mice display a shift of fast-twitch, glycolytic fibers towards slower, oxidative fibers (Seto et al., 2011). In line with these phenotypes, the *ACTN3* R577X polymorphism is underrepresented in elite sprint and power performance athletes but overrepresented in endurance athletes (Yang et al., 2009). In aged humans, the polymorphism seems to be deleterious for the maintenance of muscle performance, as suggested by the effects on walk time (Delmonico et al., 2008; Tan et al., 2012). Similarly, aged *ACTN3* knockout mice have lower grip strength and muscle mass than their wild-type counterparts, primarily due to atrophy of fast-twitch IIb fibers (Seto et al., 2011).

Polymorphisms in the angiotensin converting enzyme (*ACE*) gene, a key component of the renin-angiotensin pathway and a stress hormone that promotes muscle proteolysis (Brink et al., 2001), have also been linked to muscle atrophy and athletic performance in the young, and to muscle functional decay in the aged (Carter and Groban, 2008). Pharmacological inhibition seems to protect humans and mice from sarcopenia (Carter and Groban, 2008), but further studies are needed to better define the function of ACE during sarcopenia.

Although mammalian models are useful in these studies, the short generation time of *Drosophila* (around 10 days) could streamline the experimental selection, over several generations, of *Drosophila* strains that have increased or decreased lifespan and motor activity (Rose and Graves, 1989; Mackay, 2002; Desroches et al., 2010; Wilson et al., 2013), which could enable the identification of polymorphisms associated with age-related muscle dysfunction. Moreover, the analysis of large, genetically homogeneous populations could shed light on the epigenetic and stochastic changes responsible for individual variation in developing age-related muscle dysfunction (Grover et al., 2009). For example, polymorphisms or changes in the expression of the *Drosophila* homologs of *ACTN3* (*Actn3*) and *ACE* (*Ance* and related genes) might illuminate differences in the severity of muscle aging in distinct populations and individuals.

## Therapies for muscle aging

Although there are no approved pharmacological therapies for the treatment of sarcopenia in humans, inhibition of the myostatinactivin pathway has been proposed as a possible intervention to prevent muscle mass loss in the aged. For example, administration of anti-myostatin antibody improves exercise resistance in aged mice (LeBrasseur et al., 2009). Moreover, myostatin-null mice have increased muscle mass and might be protected from sarcopenia (Siriett et al., 2006), although they demonstrate compromised force production in comparison with controls (Amthor et al., 2007). Further studies are needed to conclusively address the role of myostatin in sarcopenia.

Resistance and aerobic exercise is a well-known intervention that improves muscle function and metabolism and delays the loss of muscle mass (Volpi et al., 2004; Nair, 2005; Wolfe, 2006). However, because exercise programs have little efficacy in restoring muscle mass in old age, exercise should be started in young and middle age. Exercise can similarly offset age-related motor dysfunction in *Drosophila* (Piazza et al., 2009), making this organism potentially useful for exercise physiology studies.

In addition to physical activity, DR might also slow sarcopenia in humans: it reduces myofiber atrophy and functional decline in rats, mice and rhesus monkeys (Altun et al., 2010; Colman et al., 2008; Payne et al., 2003). In mice, some of the gene expression changes observed in skeletal muscles during aging can be inhibited by DR (Park and Prolla, 2005). Moreover, when aging rats consumed 70% of the caloric intake of age-matched controls (Altun et al., 2010), muscle mass loss and the age-related increase in UPS components decreased. Although several mechanisms are probably involved, DR inhibits the IGF1–insulin signaling pathway and promotes the expression of autophagic genes in humans (Mercken et al., 2013). In addition, DR preserves mitochondrial function by preventing age-related oxidative damage and decline in coupling efficiency without promoting *de novo* mitochondrial biogenesis (Lanza et al., 2012; Miller et al., 2012).

Similarly, DR reduces the prevalence of flight defects in aged flies by increasing mitochondrial function and fatty-acid oxidation in the predominantly aerobic flight muscles (Katewa et al., 2012). However, DR does not seem to prevent the functional senescence of the glycolytic muscles used for walking and climbing (Bhandari et al., 2007), suggesting that DR can prevent age-related functional decay of some but not all fiber types in *Drosophila*. In addition, protein supplementation has also been shown to improve physical performance in old age in humans (Tieland et al., 2012), somewhat in contrast with the notion of anabolic resistance and benefits of DR. Additional studies in mammals and *Drosophila* are needed to better understand the mechanisms of action of exercise and DR, and to identify pharmacological interventions against sarcopenia.

## Conclusions

In this Review, we have highlighted several important similarities and differences between the age-related decay of muscle function in *Drosophila* and the sarcopenia and loss of muscle function seen with aging in mammals (Table 1). In mammals, including humans, muscle aging is influenced by muscle regeneration via satellite cells and other extrinsic factors, but the contribution of these components to sarcopenia is typically not distinguished from the role of intrinsic changes in myofibers. *Drosophila* is a postmitotic organism with no known regenerative or growth capacity of skeletal muscles in the adult. Studies in *Drosophila* have already pinpointed key cellular age-related changes in muscle and the role of stress resistance pathways in preserving muscle function during aging. These studies demonstrate the utility of *Drosophila* as a system for probing the role of signaling pathways in regulating age-related intrinsic changes in pre-existing myofibers, without any confounding effects deriving from regeneration and satellite cell function. Studies in *Drosophila* and mammals thus offer distinct advantages for investigating the mechanisms of muscle aging, and integrating insights from both experimental systems will benefit future research efforts, which could provide pharmacological targets for the development of effective therapies for sarcopenia in humans.

**Table 1.**
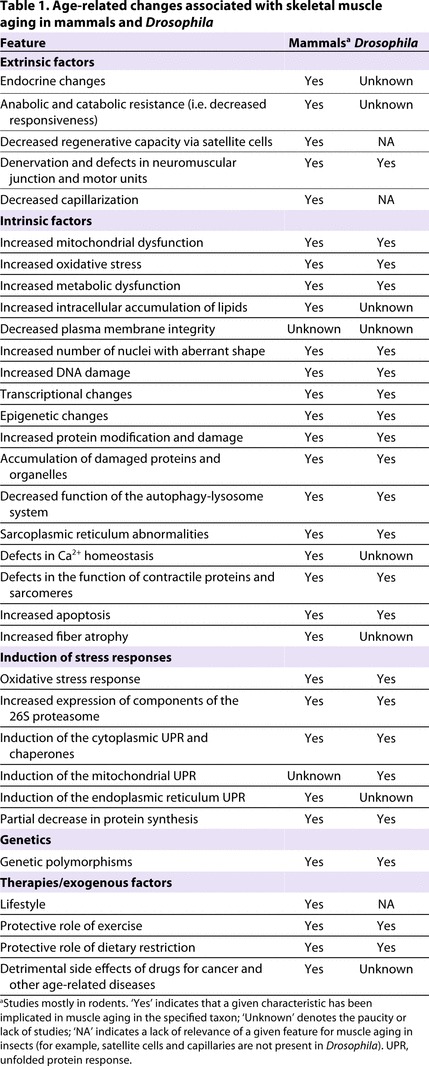
Age-related changes associated with skeletal muscle aging in mammals and *Drosophila*
